# Supplementary material for: Structural basis for high-affinity actin binding revealed by a β-III-spectrin SCA5 missense mutation
Source: Nat Commun. 2017 Nov 7;8:1350. doi: 10.1038/s41467-017-01367-w (PMC5676748; doi:10.1038/s41467-017-01367-w)
Supplement: Supplementary file 1 — Supplementary Information [file 41467_2017_1367_MOESM1_ESM.pdf]

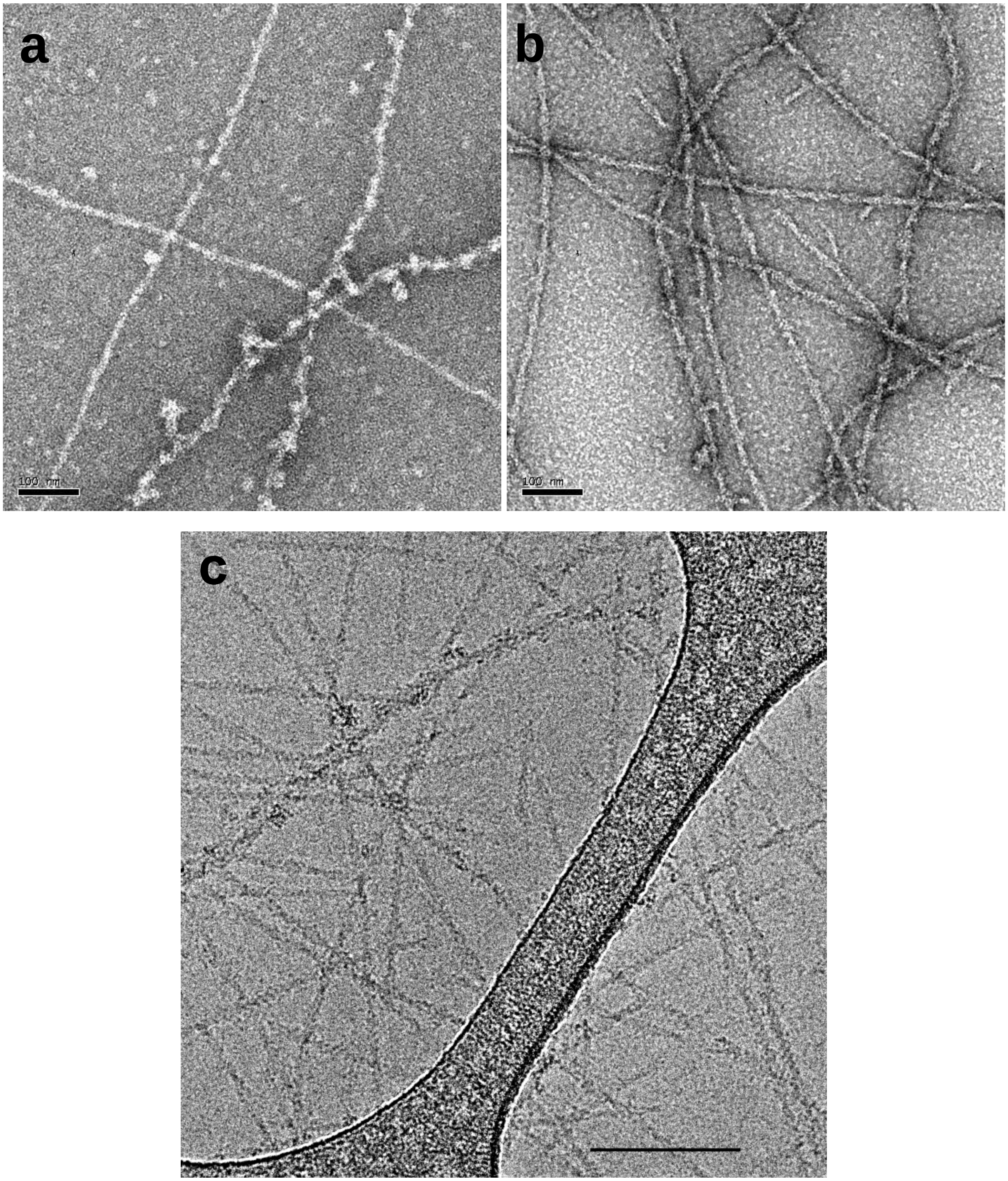

Supplementary Figure 1. Electron microscopy of β-III-spectrin ABD-actin complexes. Negative staining of (**a**) WT ABD-actin showing poor decoration of filaments and (**b**) L253P ABD-actin. Cryo-EM was used (**c**) for the 3D reconstruction of the L253P ABD decorated actin filaments. Lacey carbon grids were used, with the filaments suspended in the holes within this carbon film. The carbon film is seen near the center of the image, with holes on both sides of it. The space bars in (**a**), (**b**) and (**c**) are 1,000 Å.

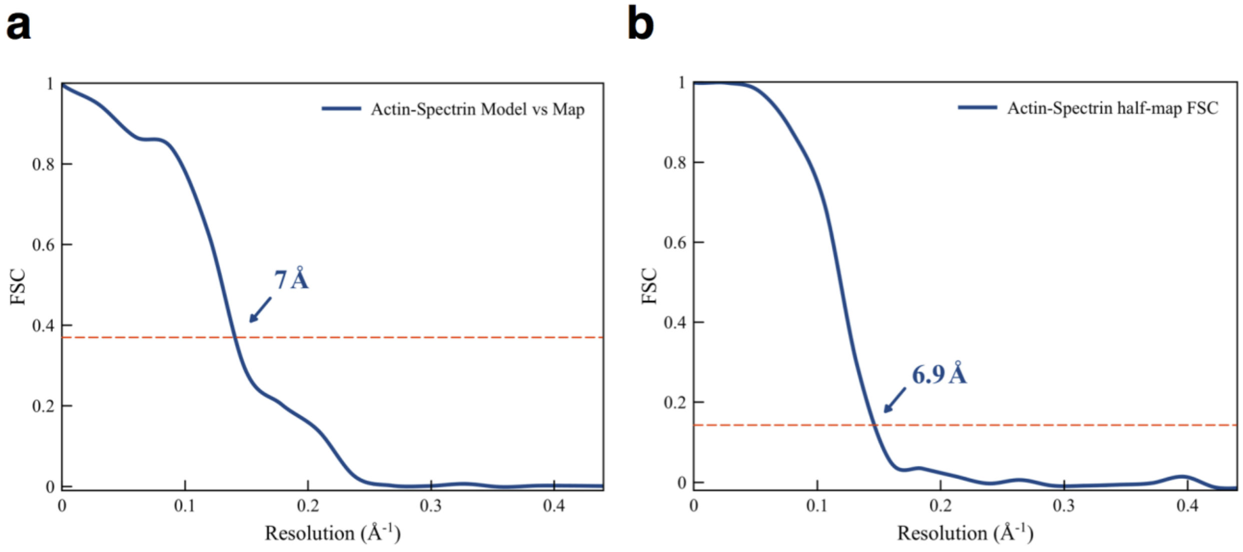

Supplementary Figure 2. Resolution of the Actin-spectrin filament reconstruction, estimated two different ways. (**a**) The resolution was derived from Fourier Shell Correlation (FSC) calculation between the refined atomic model and the map. (**b**) A "gold-standard" FSC is also calculated between two half maps. Given the relatively small number of segments used in the final reconstruction (~12k), splitting this into two halves to generate a conventional "gold standard" map:map FSC can be problematic, as the result from the half-maps is biased to a lower resolution than the full map[1]. Nevertheless, this method yielded an estimate of 6.9 Å resolution at FSC=0.143. For the map:model comparison, we used FSC=0.38 where 0.38=√0.143.

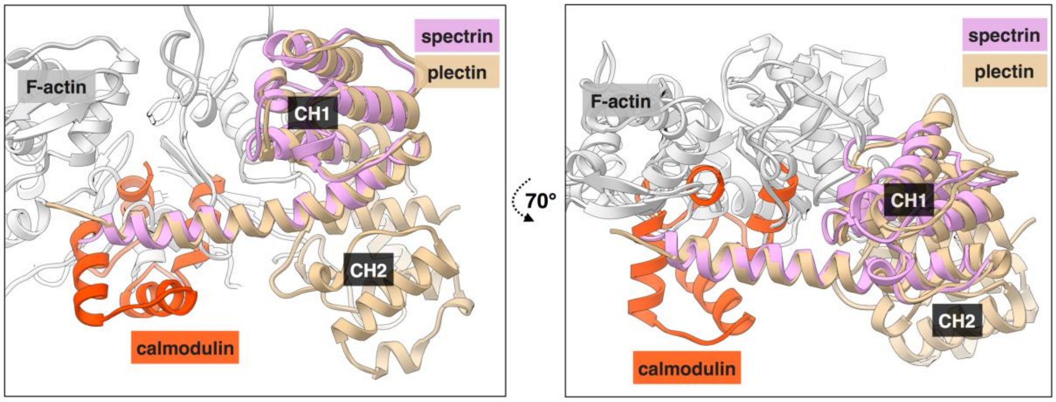

Supplementary Figure 3. Comparison with calmodulin-bound plectin structure (4Q57). F-actin (gray), β-III-spectrin (pink), plectin (brown) and calmodulin (orange) are shown. Plectin model is aligned with spectrin using only the N-terminal helices (rmsd=0.8 Å), and a major clash would result between calmodulin and F-actin.

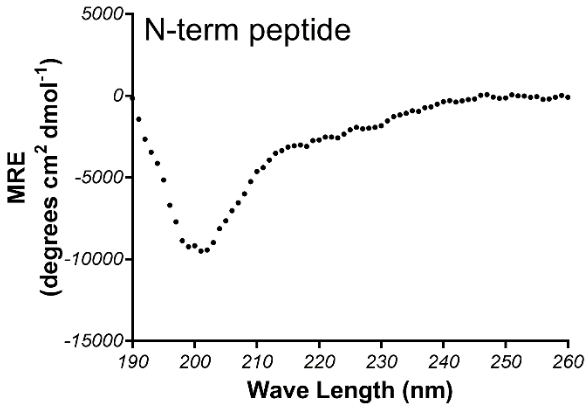

Supplementary Figure 4. Circular dichroism spectra on a peptide corresponding to the β-III-spectrin N-terminus. The absorption profile is consistent with a disordered structure.

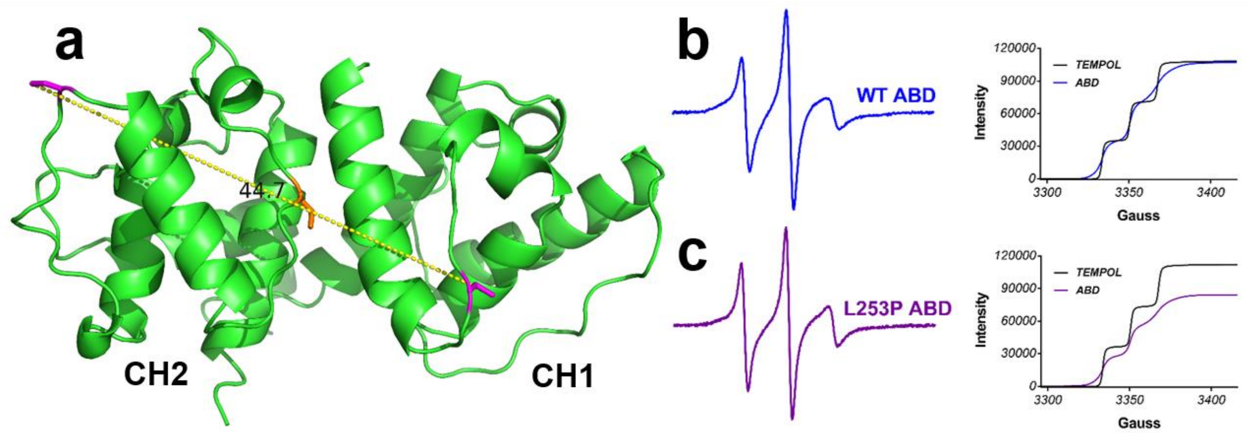

Supplementary Figure 5. Site-directed spin labeling of β-III-spectrin ABD. (**a)** Homology model generated for β-III-spectrin ABD from crystal structure of the closed state of α-actinin, indicating the approximate inter-probe distance (side chain-side chain distance of 4.5 nm, yellow dotted line) between two native cysteine residues (C76 and C231, magenta residues) and highlighting L253 in orange at the CH domain interface. (**b** and **c**) CW EPR spectrum (left) of MSL-labeled β-III-spectrin ABD and example spin count (right) for WT (blue) and L253P mutant (purple). Note probe mobility is as expected for cysteine residues located in loops.

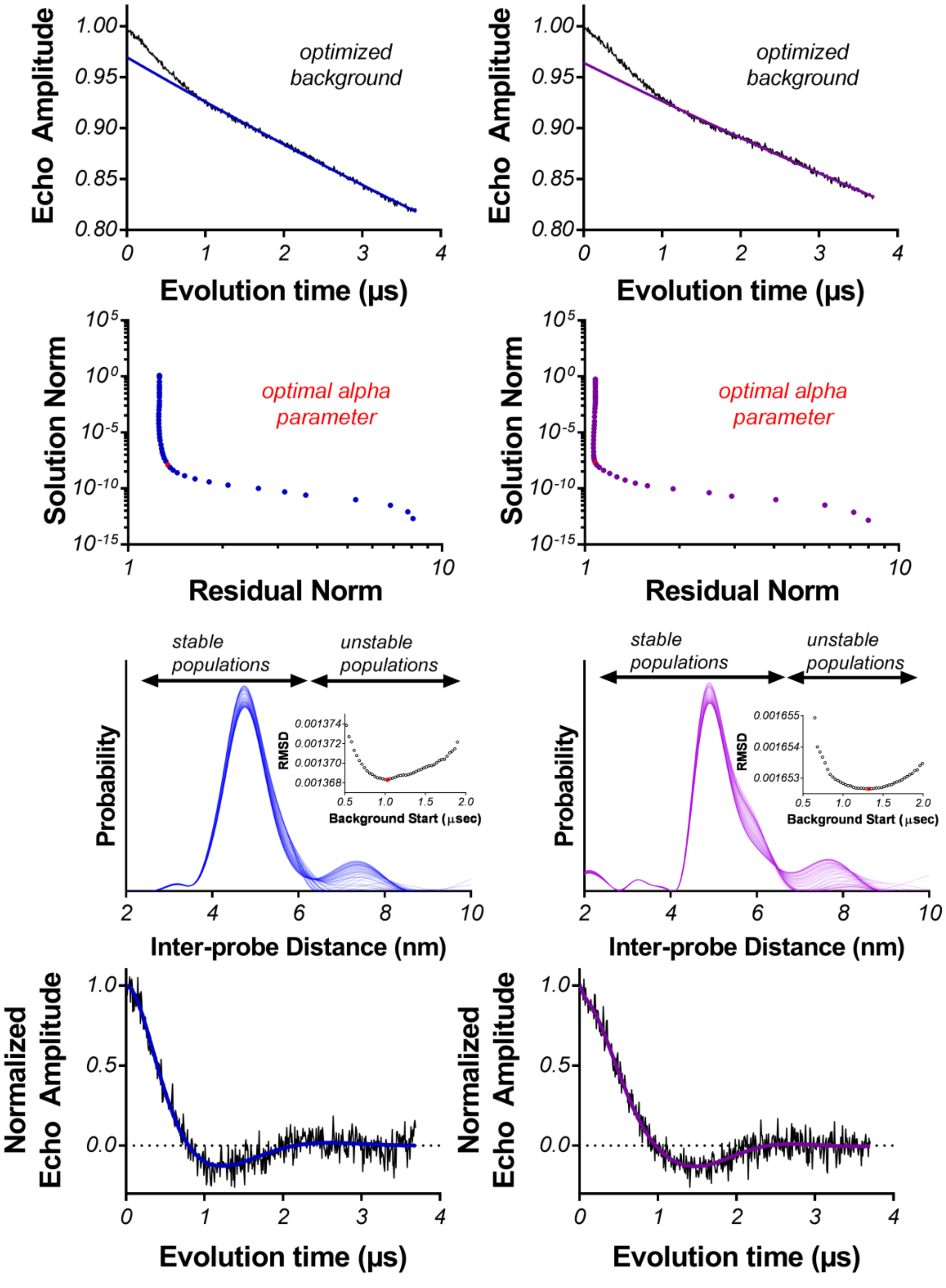

Supplementary Figure 6. DEER analysis of WT (blue) and L253P (purple) β-III-spectrin ABDs. (top row) Raw echo amplitude decays with optimal background. (middle rows) L-curve analysis indicating smoothing parameter chosen from Tikhonov fit of the

echo amplitude decay. As the distance distributions from fitting the DEER waveform are highly sensitive to the choice of the background component, we examined the impact of a range background fits (varying the start of the fit from 0.5 μsec to 2.4 μsec on the uncorrected waveform). The components that were invariable with respect to choice of background were used for structural interpretation of the ABD constructs. The component that was not stable, and in some cases disappeared entirely (the peak(s) at >7.0 nm), was excluded from structural interpretation as it represents an artifact of background subtraction. (bottom row) Normalized echo amplitude decays fit with the Tikhonov model corresponding to the lowest RMSD.

Supplementary Table 1

	Actin-spectrin filament
Clash score, all atoms	6.6
Protein geometry	
Ramachandran favored (%)	92.7
Ramachandran outliers (%)	0
Rotamer outliers (%)	0
Cβ deviations > 0.25 Å (%)	0
RMS deviations	
Bonds (Å)	0.01
Angles (°)	1.00
MolProbity score	1.82 (99th, 3.25 Å – 7.25 Å)
PDB ID	6ANU

Refinement statistics for the actin-spectrin filament model by MolProbity [2].

Supplementary References

1. Subramaniam, S., Earl, L.A., Falconieri, V., Milne, J.L. & Egelman, E.H. Resolution advances in cryo-EM enable application to drug discovery. *Curr Opin Struct Biol* **41**, 194-202 (2016).
2. Chen, V.B. et al. MolProbity: all-atom structure validation for macromolecular crystallography. *Acta Crystallogr D Biol Crystallogr* **66**, 12-21 (2010).
